# The Quality of Lunches Brought from Home to School: A Systematic Review and Meta-Analysis

**DOI:** 10.1016/j.advnut.2024.100255

**Published:** 2024-06-12

**Authors:** Siwan Song, Elizabeth Tabares, Ariun Ishdorj, Molly Crews, Jayna Dave

**Affiliations:** 1 Department of Agricultural and Applied Economics, University of Wisconsin-Madison, Madison, WI, United States; 2 Norman Borlaug Institute for International Agriculture and Development, Texas A&M University, College Station, TX, United States; 3 Department of Agricultural Economics, Texas A&M University, College Station, TX, United States; 4 School of Veterinary Medicine & Biomedical Sciences, Texas A&M University, College Station, TX, United States; 5 USDA/Agricultural Research Service Children’s Nutrition Research Center, Baylor College of Medicine, Houston, TX, United States

**Keywords:** lunches brought from home, packed lunch, nutrition, systematic review, meta-analysis

## Abstract

This systematic review and meta-analysis, spanning studies published between 1995 and 2021, investigates various aspects of lunches brought from home (LBFH) to school by children. These meals, in contrast to those provided by the National School Lunch Program (NSLP), lack strict nutritional standards. Despite the availability of NSLP lunches, ∼40% of US children opt for LBFH. This review aims to assess the food content and nutritional quality of LBFH, their adherence to NSLP standards in terms of nutrition and cost, effectiveness of intervention programs designed to enhance their nutritional quality and parental and student perceptions of LBFH. The comprehensive literature search yielded 28 eligible papers, with 16 included in meta-analysis. LBFH commonly include fruits (50%), yet vegetables (17%) and dairy (25%) are less prevalent. They frequently contain snacks (50%), sweets (48%), and sugar-sweetened beverages (31%). Compared with school lunches, LBFH exhibit lower levels of calcium, protein, iron, fiber, and vitamin A, and higher levels of carbohydrates and saturated fat. Intervention programs had no effect on quality of LBFH. On average, LBFH ($1.81) cost slightly less than lunches served at school ($1.98), without accounting for free/reduced-price meals in the calculation. The cost of school lunch for pre-k and kindergarten children became $11.32, nearly 4 times higher than that of LBFH ($2.92), after replicating the meal at home and accounting for meal preparation time. Parents preferred LBFH over school lunches because of concerns related to the quality of school meals served. This study concludes that LBFH are generally less nutritious compared with lunches provided by NSLP. Future research needs to further explore ways to improve parent perception of NSLP. Especially with many states making free meals available to all children, identifying effective ways in promoting and increasing NSLP participation can ensure more children have access to nutritionally balanced and affordable lunches.

**Statement of Significance**

This review provides quantitative and qualitative evidence that a significant proportion of school-aged children who bring their lunch from home to school consume less nutritionally balanced meals, which are biased toward having more sweets and fewer vegetables. This review contributes to a deeper understanding of the nutritional quality of these lunches, the characteristics of children who bring lunches from home, and the implications for child nutrition.

## Introduction

In the United States, school-age children often fall short of consuming the recommended amounts of fruits, vegetables, whole grains, and low-fat dairy, while frequently consuming excessive calories from energy-dense and nutrient-poor foods and beverages [1]. Such unhealthy eating patterns lead to diminished academic performance, obesity, and related chronic diseases such as type 2 diabetes, and cardiovascular diseases that track into adulthood [2]. In addition, school-age children spend the majority of their weekdays at school and children who consume National School Lunch Program (NSLP) meals, obtain a third of their daily energy needs from these meals [3]. The meals provided by the NSLP have undergone substantial improvements because of the Healthy, Hunger-Free Kids Act [4]. These improvements have resulted in more nutritious options, including increased whole-grain foods and greater portion sizes for fruits and vegetables (FVs) while limiting energy and sodium intake as well as removing foods containing trans fats. These changes have positively impacted the diet quality of children participating in the NSLP [5,6].

In fiscal year 2019, schools served over 4.8 billion NSLP lunches. Prior research has extensively documented the quality of the NSLP lunches served at school, and the amount of food and nutrients consumed from these lunches [[6], [7], [8], [9], [10], [11]]. This is evident from multiple systematic reviews that have examined the impact of the NSLP on various health and nutritional outcomes in school-age children [[12], [13], [14]].

Although many children participate in the NSLP, ∼40% of children nationwide still bring their lunches from home to school on any given day [15]. The lunches brought from home (LBFH) are less regulated and not required to meet the same meal requirements and nutrient standards of the NSLP. Therefore, it is plausible that LBFH may benefit from efforts aimed at improving their nutritional quality.

There are relatively few studies that have examined the food and nutrient content of LBFH to school, and the amount of food and nutrients consumed from these lunches [6,[15], [16], [17], [18], [19], [20], [21], [22]]. To date, no systematic review with meta-analysis has been conducted on LBFH.

To fill this gap and provide a holistic overview of the LBFH in the literature, we aim to consolidate and synthesize all the existing quantitative and qualitative evidence on LBFH by *1*) examining food and nutrient content of LBFH by children in pre-K through 12th grade by using the proportion of students who brought each food item from home as an effect size, *2*) comparing the nutrient content of LBFH to lunches served by the NSLP and the number of nutrients consumed from LBFH to nutrition standards of the NSLP by employing Hedge’s *g* as an effect size measure, *3*) examining the effectiveness of intervention programs in improving the quality of LBFH by utilizing a narrative synthesis approach, *4*) comparing the cost of LBFH to the NSLP meals, and *5*) exploring parents’ and students’ perceptions about LBFH and factors influencing their preferences for LBFH over lunches served by the NSLP.

## Methods

This systematic review and meta-analysis were conducted using the PRISMA guidelines. The protocol is registered on the PROSPERO International Prospective Register of Systematic Review (CRD42022299879).

An academic librarian specialized in systematic reviews and search synthesis conducted database searches in the EBSCO databases of MEDLINE, Agricola, ERIC, and CINAHL, as well as CAB Direct and ProQuest Theses and Dissertations; a second librarian peer-reviewed the searches. To supplement the database searches, gray literature was manually searched using search engines including Google Scholar (in an incognito browser) and Carrot2. Reference lists of systematic reviews and key included articles were manually searched to identify additional relevant studies. Database searches were conducted in December 2021 and manual searches were conducted in January 2022. Database search details are available in the Supplementary material.

### Inclusion and exclusion criteria

This review included peer-reviewed papers, policy papers, working papers, thesis/dissertation, and studies that met the following criteria: *1*) studies conducted in US schools (Pre-K through 12th grade); *2*) studies reporting food and nutritional quality of LBFH and their consumption but not necessarily the NSLP and other non-LBFH information; *3*) observational or intervention studies reporting quantification of any outcomes related to LBFH but not necessarily the NSLP other non-LBFH information; *4*) articles written in English; and *5*) studies published between 1995 and 2021. The year 1995 was chosen as the starting point because this is the year in which the School Meals Initiative was issued [23]. Between 1995 and 2021, the only significant change to school meal standards occurred in 2012. Although the Child Nutrition and Special Supplemental Nutrition Program for Women, Infants, and Children Reauthorization Act was passed in 2004, it did not mandate substantial changes to the nutrient content of school meals.

Exclusion criteria included *1*) conference proceedings or posters published online without the full article; *2*) opinions, editorials, newspaper articles, forms of popular media, background articles, and any other articles not presenting original research; *3*) articles with a wrong population, comparator, or study design; *4*) duplicated articles; and *5*) articles reporting validation of methodology.

### Study selection and data extraction

Search results were uploaded to Covidence [24], a web-based collaboration software platform that streamlines the production of systematic reviews and other types of literature reviews. Two researchers independently screened titles, abstracts and full-text articles for relevance and eligibility, and conflicts were resolved by a third researcher.

Using Google Forms and Excel spreadsheet, 2 researchers extracted data from the included studies and a third researcher reviewed the data for completeness and accuracy. The following information was extracted from each included study: study identification (first author’s name, publication year, data, and collection date), study design (type of study design, geographical location of data collection, sample size, data collection method, data collection year and duration, nutrition standards, dietary guidelines and assistance programs considered), sample characteristics (school grades); school characteristics (type of school, location, etc.), evaluated food items (fruit, vegetables, FVs combined, whole-grain, and milk) and nutrients (energy, sodium, saturated fat, vitamins, dietary fiber, and protein), a unit of measurement, Healthy Eating Index (HEI) [25] reported in the studies, and conclusions.

### Quality and risk of bias assessment

Quality assessment of included studies was performed using the USDA’s Nutrition Evidence Systematic Review (NESR) Bias Assessment Tool supported by the 2020 Dietary Guidelines Advisory Committee for evaluating study bias [26]. To assess observational studies, we used the Risk of Bias for Nutrition Observational studies (RoB-Nobs), designed for evaluating food and nutrition observational studies. The RoB-Nobs includes domains such as confounding, selection of participants, classification of exposure, departures from intended exposures, missing data, measurement of outcomes, and selection of reported results. The overall evaluation can be categorized as “low,” “moderate,” “serious,” or “critical” risk of bias, as well as “no information.” To assess randomized control trial studies (RCTs), we employed the Cochrane Risk of Bias tool for randomized trials (RoB 2.0). This tool addresses the bias arising from the randomization process, the timing of identification and recruitment of individual participants in relation to the timing of randomization, deviations from intended interventions, missing outcome data, measurement of the outcome, and selection of the reported results. The overall risk of bias in RCT studies is categorized as “low,” “some concerns,” “high,” and “very high.” For both observational studies and RCTs, the overall risk of bias was determined by the most severe level of bias found in any individual domains evaluated.

### Data analysis and synthesis strategy

A narrative synthesis approach for systematic reviews was used for the assessment of the robustness of the synthesis following the guidelines [27] and the USDA NESR Conclusion Statement Evaluation Criteria [26].

To assess the food content of LBFH, we examined the prevalence of specific food items included in the student’s lunch, such as the percent of lunches with a snack, dessert or sweets, fruit and/or vegetables, dairy food, or beverage by conducting the random-effects univariate meta-analysis by food item across all studies. The effect size for each food component was calculated using the proportion of students who brought the food item [28], and a univariate meta-analysis was conducted for each food item. Heterogeneity was evaluated using the *I*^2^ statistic, with *I*^2^ <50% is generally considered to be an acceptable level of heterogeneity and *I*^2^ exceeding 50%, indicating the presence of a substantial degree of heterogeneity among studies. In addition, the HEI-2010 was used as a measure of diet quality assessment in some of the studies included in this review and HEI scores were extracted from these studies to summarize the findings. The HEI consists of 12 components. Nine of these components focus on adequacy emphasizing the consumption of total fruit, total vegetables, greens and beans, whole grains, dairy, total protein foods, seafood and plant proteins and fatty acids. The remaining 3 components are designed for moderation, aiming to limit the consumption of refined grains, sodium and empty calories [29]. HEI scores range from 0 to 100, with a higher score indicating better diet quality. The extracted HEI scores were classified into 3 categories. A HEI score from LBFH > 80 was classified as a “good” diet, a score between 51 and 80 was classified as a diet that “needs improvement,” and a score < 51 was classified as a “poor” diet [25].

To examine the nutritional content of LBFH we used energy, total fat, saturated fat, carbohydrates, protein, iron, calcium, fiber, vitamins A, and C, and sodium in both LBFH and lunches provided by the NSLP. Hedge’s *g* [30] was calculated by taking the difference of the mean of energy and each nutrient value between LBFH and school lunches, divided by the pooled standard deviation, and including a correction factor to account for potential bias because of small sample sizes. Hedge’s *g* values of 0.2, 0.5, and 0.8 were considered small, moderate, and large in magnitude, respectively. The meta-analyses used random-effects modeling. We further interpreted Hedge’s *g* using common language effect sizes, translating the effect sizes into probabilities for a better understanding of the results.

In addition, we examined the amount of energy, macronutrients, and micronutrients (vitamins and minerals) consumed from LBFH and compared them with nutrition standards. Specifically, to assess energy, saturated fat, and sodium consumption from LBFH we compared the amount consumed from LBFH to the NSLP standards. To examine carbohydrates, protein, iron, calcium, fiber, vitamin A, vitamin C, and total fat consumption from LBFH, we compared the amount consumed from LBFH to the 33% Recommended Dietary Allowance (RDA). Here we are assuming that children are consuming a third of their daily food intake at lunch, especially if they are participating in the NSLP [3, [31], [32], [33]].

To assess the effectiveness of intervention programs designed to improve the nutritional quality of LBFH, we employed a narrative synthesis approach.

To investigate the cost of LBFH, we conducted a comprehensive review of relevant studies, synthesizing data related to food prices and costs associated with preparing LBFH and replicating the NSLP lunches at home.

Lastly, the perceptions of parents and students regarding choosing LBFH over lunches provided by the NSLP was investigated by examining the relevant qualitative data and conducting research synthesis.

## Results

### Search results

**FIGURE 1:**
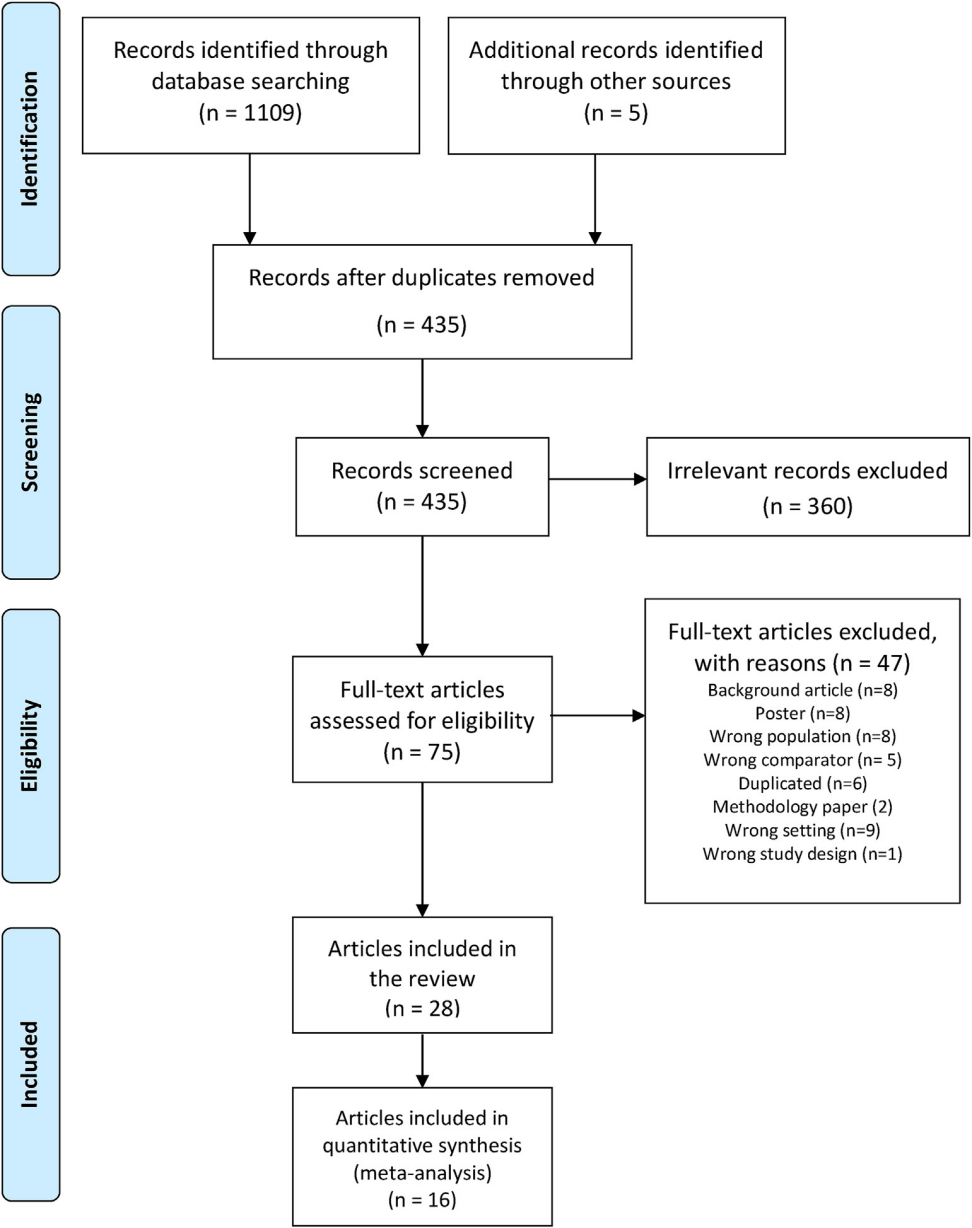
PRISMA flow diagram.

The electronic search retrieved 1109 records from databases and the manual searches of reference lists and other sources identified an additional 5 records. After removing duplicates, we screened 435 titles and abstracts and removed 360 records as irrelevant (Figure 1). Subsequently, a full-text screening was conducted of the 75 articles, excluding 47 articles for various reasons, such as not meeting inclusion criteria for study type (for example, background articles), publication type (for example, conference posters), and study population and design. A total of 28 articles (21 peer-reviewed publications, 1 PhD dissertation, and 6 MS theses) were selected which were merged into 18 unique studies based on their data sources. Articles that used data collected from the same population, overlapping populations or subsets thereof were merged into 1 study. Similarly, dissertations, theses and/or peer-reviewed publications from the dissertation or thesis were merged into a single study. The included studies contained data from 18,912 children from studies conducted in 13 states with the sample size ranging from 31 to 2107 children. Texas and California had the highest number of studies (*n* = 3). The 11 articles that provided the information needed to calculate the effect sizes were used in the meta-analysis.

Among the 18 independent studies (published in 28 articles), 16 were observational, 1 was an intervention, and 1 was an exploratory interventional study consisting of 5 published articles that provided both baseline and intervention results (Table 1 [[15], [16], [17], [18], [19], [20], [21],[31], [32], [33], [34], [35], [36], [37], [38], [40], [41], [42], [44], [45], [46], [47], [48], [49], [50]]). The sample sizes of the included studies ranged from 31 to 2107 children. These studies were conducted in various settings, pre-k through elementary schools (*n* = 1), both kindergarten and elementary schools (*n* = 2), kindergarten through middle schools (*n* = 1), elementary schools (*n* = 12), middle school (*n* = 1), and high school (*n* = 1). Four primary data collection methods were used: 24-h recall (*n* = 3), direct observation (*n* = 10), digital photography (*n* = 3), and a combination of direct observation, survey and/or digital photography (*n* = 2).

**TABLE 1:** Characteristics of included studies

Study and publications	Location	Data collection method	Study population (grade/age)	Number of observations	Outcome	Data collection year	Standards or programs considered	Main results	Risk of bias
Observational studies									
Neubauer (2000) [31]	New York	24-h dietary recall	Elementary (third)	31	Nutrients brought	2000	Recommended Dietary Allowances	LBFH were analyzed for energy, protein, vitamins A, C, and B-6, calcium, iron, zinc, sodium, and cholesterol. All the nutrients examined were insufficient, especially vitamin A and zinc. Findings showed low occurrences of fruits and vegetables and high incidence of sugar and salty foods.	Critical
Hamilton (2001) [16]	West Virginia	Direct observation	Elementary (second–fifth)	114	Nutrients brought and consumed	1996	NSLP	There was a statistically significant difference in nutrient content between school lunches and LBFH for energy available as protein and sugar, dietary fiber, vitamin A, zinc, the bone-related nutrients, and the energy facilitating nutrients.	Critical
Rainville (2001) [34]	Michigan	Direct observation	Elementary (second–fourth)	570	Nutrients brought	1997	NSLP	Reimbursable school lunches contained significantly fewer calories from fat (29%) than LBFH (33%), significantly more protein, fiber, vitamins A, D, B6, B12, thiamin, riboflavin, niacin, folate, calcium, iron, and zinc than LBFH.	Serious
Conway et al. (2002) [35] 1	California	Direct observation	Middle (sixth–eighth)	1381	Food item and nutrients brought	1997	None	The most common LBFH components were beverages and sandwiches. Fruits were more common than vegetables; non-chip snacks and chips were more common than cookies, candy, and cakes/pies. LBFH averaged 596.2 kcal, 20.8 g of total fat, 6.2 g of saturated fat, 32.6 mg cholesterol, and 21.3 g of sugar. Boys’ LBFH had significantly more kilocalories, total fat, saturated fat, cholesterol, and percentage of energy from fat than girls’ LBFH.	Moderate
Zive et al. (2002) [36]					Fat	1997	NSLP, a la carte sales, Student store sales	LBFH included 6.2 grams of saturated fat and 20.8 grams of total fat, on average.	Critical
Evans (2007) [37]	Illinois	Direct observation	Elementary (third–fifth)	34	Nutrient brought, Food items	2007	NSLP standards	The most common component of LBFH was a sandwich, observed in 90% of the lunches. Lunches containing fruits were more common (64%) than those containing vegetables (11%). Chips and snacks were seen more often than cookies or candy. 76% of lunches included a beverage. A key nutrient recommendation never met under any analysis was calcium. Females met more nutrient standards overall when vs. males.	Critical
Lund (2008) [38]	Minnesota	Direct observation	Elementary (first, third)	1555	Nutrients brought	2000	NSLP	Student eating school lunches had significantly higher intakes of protein, calcium, cholesterol, sodium, vitamin A and iron; while LBFH had significantly higher intakes of carbohydrates, fats, and vitamin C.	Critical
Doughty (2009) [17]	Connecticut	Digital photo	High (Age 14–18)	56	Nutrients consumed	2009	NSLP	Compared to LBFH, school lunches were lower in calories, higher in protein, calcium, and iron, and lower in carbohydrates, fiber, sugar, fat, saturated fat, vitamin A, vitamin C, and sodium. Significant differences were found for sugar and calcium only.	Serious
Johnson et al. (2009) [32]	Texas	Direct observation	Elementary (K-5, K-6)	317	Food item brought, Frequency of common home-packed lunch items, Costs	2006	USDA nutrient standards, NRC recommendations, Dietary Guidelines 2005 recommendation	The mean nutrient content of LBFH differed significantly from NSLP and other nutrient standards for several nutrients. LBFH contained less than recommended amounts of calories, vitamin A, calcium, iron, and dietary fiber; they contained higher than recommended amounts of total fat, protein, vitamin C, and sodium. LBFH met NSLP standards for saturated fat.	Serious
Warren (2010) [39]	New York	Direct observation	Elementary (second)	94	Nutrients brought	2010	SMI Standards	All school lunches exceeded SMI standards for calories, protein, and fat. LBFH, on average, met SMI standards for fat, but exceeded calorie and protein standards. School lunches contained significantly more calories, protein, and fat than LBFH.	Serious
Hur et al. (2011) [40]	Minnesota	Direct observation	Elementary (fourth–fifth)	129	Nutrients brought	2005	NSLP, SMI Standards	LBFH included significantly more calories, total fat, and total sugar than NSLP lunches using SMI standards. LBFH contained slightly more dietary fiber (4.1g vs. 3.8g), and significantly less sodium (728 mg vs. 912 mg). LBFH were significantly lower in fruits and vegetables.	Serious
Johnston et al. (2012) [15]	Texas	Direct observation	Elementary (second)	2107	Food item brought, % contents of each lunch type	Not specified	NSLP, SMI Standards	LBFH contained significantly fewer fruits (45.3% vs. 75.9%), vegetables (13.2% vs. 29.1%), dairy (41.8% vs. 70%) and more desserts (60% vs. 17.5%) and sugar-sweetened beverages (SSB, 47.2% vs. 0.3%) than NSLP lunches.	Moderate
Bergman et al. (2014) [18]	Washington	Digital photo	Elementary (second–fifth)	620	Nutrients brought and consumed	2011–2012 academic year	NSLP, SMI Standards	School lunches were significantly higher in protein, cholesterol, calcium, iron, sodium, and vitamin C than LBFH. LBFH included significantly less calories, more fat and saturated fat than NSLP lunches. Students who consumed NSLP lunches were significantly more likely to meet SMI standards for percentage of calories from fat, protein, calcium, iron and vitamins A and C. Students with LBFH consumed significantly more saturated fat, fiber, and less sodium than students who consumed NSLP lunches.	Moderate
Bergman et al. (2016) [19]				1033	Food item and nutrient brought and consumed, HEI	2011–2012 academic year	NSLP	LBFH provided significantly more Whole Grains and Seafood & Plant Proteins than NSLP lunches. NSLP lunches scored higher in Total Protein and reduced Empty Calories. Selection of 1% plain milk resulted in significantly higher HEI-2010 scores.	Moderate
Caruso and Cullen (2015) [20] 1	Texas	Direct observation	Elementary, Intermediate (K-eighth)	334	Food item brought and consumed	2011	2012 NSLP Nutrition standards	90% of LBFH included desserts, snack chips and SSB. LBFH contained significantly more sodium, less vegetables, and exceeded saturated fat limits vs. NSLP nutrition standards. Intermediate school students’ LBFH did not contain adequate amounts of vegetables, fruits, whole grains, and milk (brought or consumed) when vs. the NSLP nutrition standards.	Serious
					Costs	2011	None	The cost of LBFH averaged $1.93 for elementary and $1.76 for intermediate students. Students from lower-income intermediate schools brought significantly higher-priced ($1.94) lunches than did students from middle-income schools ($1.63).	
Song et al. (2021) [33]					Food item and Nutrient brought and consumed	2011	2012 NSLP Nutrition standards, DRI	Almost all LBFH contained grain and meat/meat alternatives, and the amount brought and consumed exceeded the NSLP standards. Most students did not bring fruits, vegetables, and whole-grain foods, but those who brought consumed most of what they brought. Among elementary school students, only 9% of boys and 14% of girls brought vegetables and the amount brought and consumed did not meet the standards. Although carbohydrate and protein consumption were adequate for boys and girls, the intakes of micronutrients and fiber did not meet the requirements across both genders at both school levels.	Critical
Au et al. (2016) [41]	California	24-h dietary recall	Elementary (fourth–fifth)	662	HEI	2011–2012 academic year	NSLP	School lunches had higher overall diet quality score compared with LBFH. Children who ate school lunches had higher scores for dairy and for empty calories from solid fats and added sugars.	Moderate
Nadaud (2018) [42]	Maryland	24-h dietary recall	K, elementary (first grade, Age 5–8)	71	Healthy eating index and similar, food components for HEI	2016–2017 academic year	None	Parents pack a lunch for their child because they believe that school meals are unhealthy and that their child would not like it. The mean HEI-2010 score was 63.9. Children’s Total vegetable scores and Whole fruit scores tended to increase with their parents’ self-efficacy for enacting healthy diet behaviors in their children. Added sugars scores significantly increased with their parents’ self-efficacy for enacting healthy diet behaviors in their children.	Serious
					Perceptions			The main reasons for not buying school lunches are that parents preferred to choose what their child eats (53%), the food served is unhealthy (49%) and their child would not eat the school lunch (40%). In addition, 80% of parents reported that LBFH preparation was not difficult. The unadjusted bivariate analysis showed that self-efficacy for preparing a healthy LBFH in difficult situations was negatively correlated with parents perceived constraints of LBFH and self-efficacy for enacting healthy diet behaviors in their children was rated higher with higher levels of parental monitoring of child’s eating. The more parents perceived their child’s peers influenced their child’s food choices, the more they perceived that LBFH have constraints, showed restrictive attitude, and tended to pressure their child to eat. The adjusted analysis showed that parents perceived more benefits in LBFH when their self-efficacy for enacting healthy diet behaviors in their children was rated higher. Also, Asian parents perceived more benefits in LBFH vs. Caucasian parents.	
Sutter et al. (2019) [43]	California	Direct observation and digital photo	Elementary (fourth–sixth)	90 (parent-children pairs)	Food item brought, Number of days for packing specific food item	2013		There is a relationship between higher nutrition knowledge and behavior of packing more fruit across the week and more vegetables on Monday. Parents who are more likely to be authoritative packed fewer vegetables on Monday, but more servings across the week. Financial stress was associated with higher rates of never packing vegetables and when vegetables were packed including fewer servings, while child involvement in lunch decisions was related to packing more fruits across the week, packing vegetables on more days and more servings of vegetables on Monday.	Moderate
Taylor et al. (2019) [21]				1421	Food item brought, Food item consumed, coefficients of logistic regression	2016	NSLP	Students having LBFH were significantly less likely to have and to consume fruits and vegetables, compared with students having school lunches.	Serious
Exploratory interventional studies									
Farris et al. (2014) [44]	Virginia	Direct observation	Pre-k, K	1314	Nutrients brought, % students of food items	Not specified	NSLP	LBFH were significantly higher in calories, total fat, saturated fat, sugar, vitamin C and iron, and lower in protein, fiber, vitamin A and calcium.	Moderate
Farris et al. (2015) [45]				561	Food item brought	2011–2012 academic year	NSLP	LBFH were less likely to contain fruits, vegetables, and milk than NSLP meals and included more chips, crackers and SSB. Students whose lunch included an SSB were significantly more likely to have a lunch with less protein, fiber, sugar, calcium, and iron. Although LBFH included more processed foods and snacks, surprisingly they contained significantly less sodium than NSLP lunches.	Moderate
Farris et al. (2016) [46]		Survey	Elementary	516	Frequency of NSLP or LBH, Perception (motivating factors for NSLP, motivating factors for LBH)	Not specified	NSLP	The 2 most frequent motivational factors for NSLP participation across all schools were convenience and saving time through participation. The most frequent motivators were variety of foods, nutritional quality, and providing organic or sustainable foods for lower FRL eligibility schools), while factors for higher FRL schools were child pickiness, variety of foods, and nutritional quality	Serious
O'Keefe et al. (2020) [47]		Direct observation	Pre-k, k	1289	Food cost, time cost, full cost	2012–2013 academic year	NSLP	Statistically significant differences in median food costs were found between LBFH and school lunches. The LBFH provided a statistically significant reduction in food cost vs. NSLP. When preparation time (full cost) was considered, homemade LBFH cost the most: NSLP ($2.15), convenience LBFH ($2.56), homemade LBFH ($2.92), and replicated school lunches ($11.32). Seventy-6% (414 of 545) of LBFH contained sugar-sweetened beverages and/or dessert food items, accounting for 20% of the food cost of all observed LBFH.	Serious
Farris (2015; Chapter 5) [45] 1		Direct observation, Questionnaire, Diagnostics from Facebook	Pre-k, k	88	Food item brought	2014–2015 academic year	PACK-IT (PrepAring Complete lunches for KIds Together)	Significant differences were observed for participation in social media among the intervention groups (p < 0.01). No significant difference was observed for child feeding practices or packed food records.	High
Intervention studies									
Hubbard et al. (2014) [48]	Massachusetts	Digital photo	Elementary (third–fourth)	626	Food item brought	2011	Great Taste Less Waste/Baseline (GREEN Project) 2012 NSLP Nutrition standards	The common items of LBFH were water, a sandwich, and a snack food. Only 3% of lunches included milk, and 11% of students stated that they planned to buy milk. Forty-two% of lunches contained salty snack foods; 28% had a dessert; 34% had fruit; and 11% had vegetables. Only 27% of lunches met 3 or more NSLP standards for protein, grains, fruit, milk, and vegetables.	Serious
Goldberg et al. (2015) [49] 1				582	Food item brought	2011–2012 academic year	*Great Taste, Less Waste/Food 2 Choose*	No significant differences were observed between groups in change in mean servings or change in prevalence of items after intervention. There were no significant packaging differences.	Some concern
Blondin et al. (2021) [50]				502	Energy content of foods and professing levels of foods	2012–2013 academic year	*Great Taste, Less Waste*	Most of the LBFH was highly processed. Snack foods and desserts contributed the greatest percentage of total energy to the highly processed category at baseline and follow-up (72% and 69%). There were no significant differences from pre- to post-intervention in the intervention group for the percentage of energy brought from highly processed foods in adjusted models or any other processing level.	Some concerns

Abbreviations: DRI, Dietary Reference Intakes; FRL, free and reduced lunch; HEI, Healthy Eating Index; LBFH, lunches brought from home; NSLP, National School Lunch Program; RCT, Randomized Control Trial; SMI, School Meals Initiative; SSB, sugar-sweetened beverage.

1 Indicates RCT studies.

The quality of each of the articles was assessed using the 2020 NESR Bias Assessment Tool. Among the 25 observational articles, 9 were graded as having a moderate overall risk of bias, 10 were graded as having a serious risk of bias, and 6 having a critical risk of bias. Among the 3 RCTs, 2 were identified as having some concerns, while 1 was graded as high risk of bias.

### Food and nutrient content and quality of LBFH

#### Food content of LBFH

Nine articles examined the food content of LBFH and were used in meta-analyses [15, 19, 21, 32,33,35,37,44,48]. The results from the meta-analyses of food items in LBFH to school indicate that about half of the children brought fruits (95% confidence interval [CI]: 39.5, 60.1) (Table 2). In contrast, only 16.7% brought vegetables (95% CI: 6.9, 26.4). Dairy items were brought by 25% (95% CI: [14.1%, 35.6%], *P* < 0.001) of children. Nearly half of the children brought snacks (95% CI: [43%, 56.7%], *P* < 0.001), desserts and sweets, including cookies, baked goods (that is, cakes, muffins), pudding, and candies (95% CI: [37.3%, 59.3%], *P* < 0.001). The least common beverage choices were 100% juice and milk, both appearing in only 10% of LBFH. In contrast, sugar-sweetened beverages (SSBs) were more common in LBFH than other beverages (30.5%). There was a significant between-study heterogeneity observed for food subgroups (*I*^*2*^ = [77.9%, 98.8%], *P* < 0.001).

**TABLE 2:** Meta-analysis of food prevalence in lunches brought from home

Component	Number of articles	Overall effect size (*P* value)	*I*^2^	95% confidence interval
Snacks	6 (33–38)	0.498 (0.00)	91.33	[0.43, 0.567]
Desserts and sweets	7 (15, 33, 35–38)	0.483 (0.02)	97.75	[0.373, 0.593]
Fruit	7 (15, 21, 34–38)	0.498 (0.01)	97.73	[0.395, 0.601]
Vegetables	7 (15, 21, 34–38)	0.167 (0.01)	98.8	[0.069, 0.264]
Dairy foods	4 (15, 33, 35, 37)	0.25 (0.01)	97.54	[0.141, 0.358]
100% Juice/Not sugar added juice	5 (15, 19, 33, 37, 38)	0.107 (0.00)	77.99	[0.08, 0.133]
Milk	6 (15, 19, 33, 34, 37, 38)	0.108 (0.00)	95.19	[0.06, 0.156]
Sugar-sweetened beverages	5 (19, 33, 34, 37, 38)	0.305 (0.02)	98.45	[0.159, 0.451]

Three articles used the HEI-2010 to assess the quality of LBFH [19, 41,42]. The HEI scores reported in these studies ranged between 51.1 and 63.9. Two out of 3 studies reported HEI scores that were categorized as “need improvement” in terms of diet quality, and 1 study with HEI score categorized as “poor” diet quality. As reported in Supplemental Table 1, low scores for important HEI components such as vegetables, whole grains and seafood, and plant-based proteins contributed to the low overall HEI scores.

#### Nutrient content of LBFH compared with the NSLP

Seven articles examined the energy and nutrient content of LBFH and compared it with the nutrient content of lunches offered by the NSLP [[16], [17], [18],^34^,^36^,^40^,^50^]. The results of the univariate meta-analyses indicate that the NSLP lunches contain significantly higher amounts of calcium, protein, iron, fiber, and vitamin A compared with LBFH (Table 3). For instance, the NSLP lunches were 79% (Hedge’s *g* = 0.85, *P* < 0.001) higher in mg calcium content than that in LBFH, potentially because of the inclusion of dairy products and foods in school lunches. Conversely, the NSLP lunches had lower levels of energy, total fat, saturated fat, carbohydrates, and vitamin C. Specifically, the NSLP lunches contained 73% less saturated fat (*g* = –0.65, *P* < 0.001) measured in percentage and 66% fewer grams of carbohydrates (*g* = –0.37, *P* < 0.001) than LBFH. Heterogeneity was substantial and significant with *I*^*2*^ exceeding 90% for all nutrients except for energy, macronutrients, and micronutrients (vitamins and minerals), indicating a high degree of variation across studies because of heterogeneity rather than chance. Therefore, prediction intervals were calculated which were wider than the corresponding CI and containing both negative and positive values for all nutrients, except energy, indicating that the true effect in future studies may exhibit considerable variations [51].

**TABLE 3:** Meta-analysis of nutrients in lunches brought from home compared with National School Lunch Program

Nutrient	Number of studies	Overall effect size (*P* value)	*I*^2^	95% confidence interval	Prediction interval
Energy (kcal)	6 (16–18, 41–43)	–0.20 (0.05)	0.00	[–0.30, –0.09]	[–0.36; –0.03]
Total Fat (g)	6 (16–18, 41, 42, 44)	–0.52 (0.00)	99.49	[–1.60, 0.57]	[–5.13; 4.09]
Saturated Fat (%)	6 (16–18, 41, 42, 44)	–0.65 (0.00)	99.43	[–1.67, 0.36]	[–3.56; 2.25]
Carbohydrates (g)	5 (16–18, 41, 42)	–0.37 (0.04)	64.17	[–0.58, –0.16]	[–1.03; 0.30]
Protein (g)	5 (16–18, 41, 42)	0.78 (0.00)	92.47	[0.32, 1.23]	[–0.93; 2.48]
Iron (mg)	5 (16–18, 41, 42)	0.34 (0.00)	94.30	[–0.17, 0.85]	[–1.61; 2.29]
Calcium (mg)	5 (16–18, 41, 42)	0.85 (0.00)	96.92	[0.12, 1.59]	[–1.20; 3.70]
Fiber (g)	5 (16–18, 41, 42)	0.15 (0.00)	90.94	[–0.25, 0.55]	[–1.33; 1.64]
Vitamin A (mug)	5 (16–18, 41, 42)	0.45 (0.00)	79.55	[0.18, 0.72]	[–0.50; 1.40]
Vitamin C (mg)	5 (16–18, 41, 42)	–0.18 (0.00)	91.69	[–0.60, 0.24]	[–1.74; 1.39]
Sodium (mg)	5 (16–18, 41, 42)	0.39 (0.01)	49.15	[0.22, 0.56]	[–0.11; 0.89]

#### Nutrients consumed from LBFH compared with nutrition standards

**FIGURE 2:**
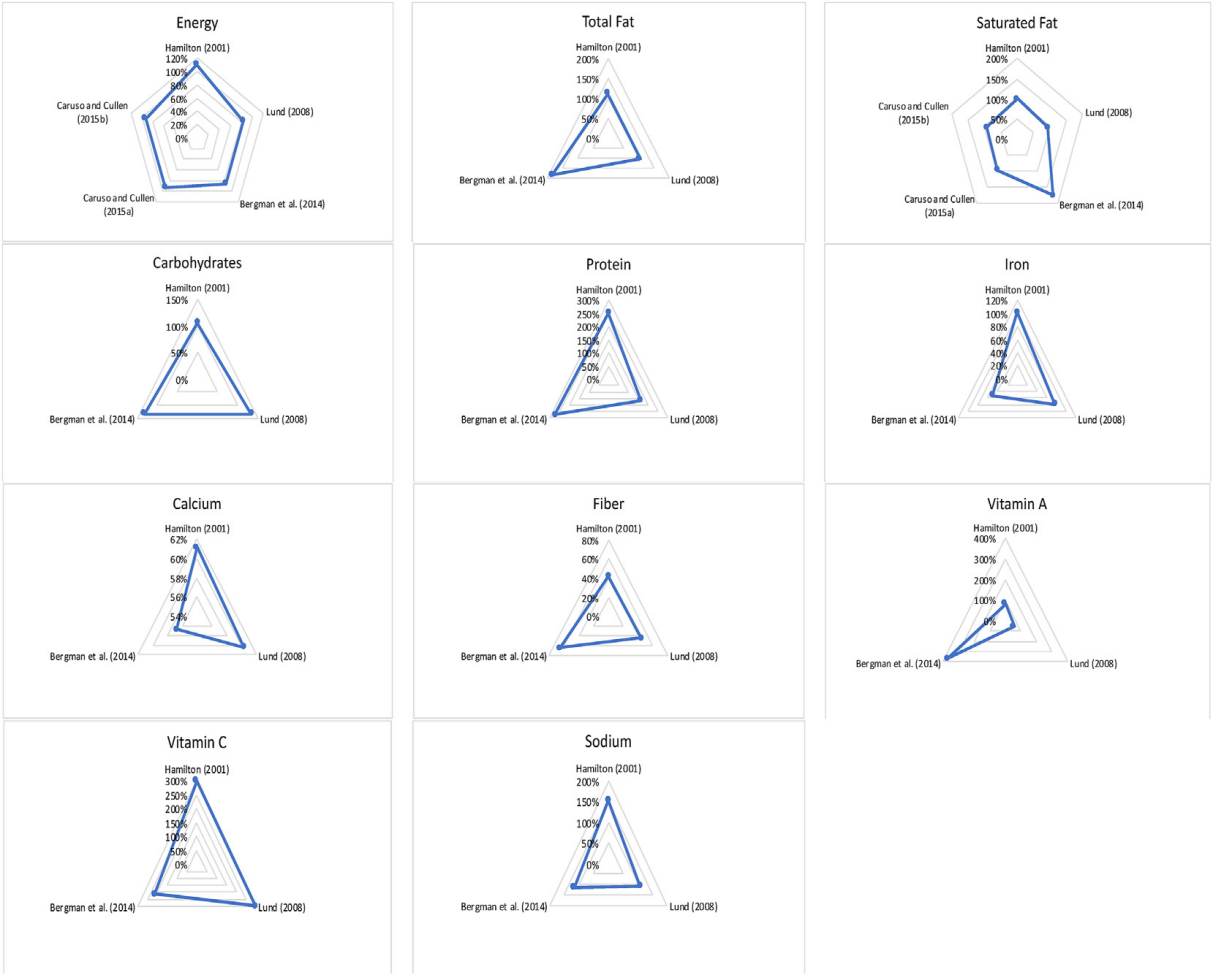
Nutrients consumed in LBFH compared with the NSLP standards. LBFH, lunches brought from home; LBFH, National School Lunch Program.

Four articles examined the amount of calories, nutrients, vitamins and minerals consumed from LBFH [16,18,20,38]. We compared the amount consumed with LBFH to school meal standards and 33% of RDA [[31], [32], [33]] and our findings reveal mixed results. As shown in Figure 2, the calories intake from LBFH fell slightly below the NSLP standards for the majority of the articles, whereas the amount of saturated fat and sodium consumed from LBFH exceeded the levels required by the school meal standards. The amount of carbohydrates, protein, vitamin C, and total fat consumed from LBFH exceeded 33% of RDA. However, the amount of iron and vitamin A consumed from LBFH surpassed 33% RDA in 2 out of 3 articles.

#### Intervention studies for LBFH

Three articles assessed the effectiveness of intervention programs designed to improve the quality of LBFH to school by school-age children [45,49,52]. *Great Taste, Less Waste*, and *Foods 2 Choose* campaigns [49] primarily targeting third- and fourth-grade children was conducted as a cluster-randomized trial in Eastern Massachusetts elementary schools during the 2011–2012 academic year. The *Great Taste, Less Waste* campaign aimed to improve the nutritional quality of children’s food choices by emphasizing connections with the environment, whereas *Foods 2 Choose* solely focused on promoting nutrition. The study compared the food content of *Great Taste, Less Waste* (*N* = 327) to *Food 2 Choose* (*N* = 78) and to the control group (*N* = 177). Lunch and snack items, including FVs and SSBs brought from home, were measured at baseline and after 7 mo using digital photography. The result showed no significant differences in the mean servings of FVs or SSBs between the groups, and there were also no differences in the prevalence of specific food items or packaging types.

The "PACK-IT" study [45] focused on elementary school students’ parents and used nutrition education and parenting strategies as interventions. The primary objective was to increase the consumption of FVs while reducing the intake of SSBs, desserts, and salty snacks in LBFH. In addition, the study examined the impact of nutrition education with social media support on lunch quality. A non-randomized quasi-experimental design was used, with parents divided into 2 intervention groups and a control group. One intervention group attended weekly nutrition education sessions and joined a PACK-IT Facebook group focused on packed lunch choices. The other intervention group attended the same sessions but joined a General Facebook group discussing various health topics. The contents of LBFH were recorded at 4 different time points corresponding to a child feeding practices questionnaire. A total of 22 parents (7 in intervention arm 1, 8 in intervention arm 2, and 7 in the control group) completed the study. Results showed significant differences in participation in social media among intervention groups but did not find significant changes in child feeding practices or the nutritional quality of LBFH. Overall, the evidence from 2 existing studies indicates that interventions designed to improve the quality of LBFH to school have not been effective [45,49,52].

#### Cost of LBFH

Only 3 articles [20,32,47] from the studies included in this review reported on the pricing and costs associated with different sources of lunches. These studies compared the average and median costs associated with various types of lunch meals, including the NSLP and LBFH, replicated school lunch and convenience-packed lunch options (for example, Lunchables). The meal costs were analyzed by grade levels, including pre-K, kindergarten, elementary, and intermediate schools. The studies compared the cost to families to prepare lunch at home to send to school compared with the cost of purchasing an NSLP lunch at school. In this comparison, none of the studies accounted for free/reduced-price meals served to the NSLP participants when calculating NSLP meal costs.

The 2 articles [20,32] that analyzed the costs of LBFH found that, on average, LBFH ($1.81) tend to be slightly less expensive than the NSLP lunches ($1.98), without accounting for free/reduced-price lunches served to over 70% of NSLP participants. In addition, another study conducted a comprehensive cost analysis, factoring in both food costs and the monetary value of labor for preparing LBFH [47]. The analysis considered the NSLP, LBFH, replicated school lunches, and convenience-packed lunches of pre-K and Kindergarten children. When considering the costs of meal preparation time, all lunch types became significantly more expensive than the lunches served through NSLP, indicating that time constraints could be a significant barrier in preparing healthy lunches. For example, the cost of a school lunch prepared at home rose to $11.32, nearly 4 times higher than that of LBFH ($2.92), after replicating the NSLP meal at home.

#### Parent and student perceptions of LBFH

Two studies examining parents’ and students’ perceptions of LBFH [42,46] and parts of intervention studies were identified and included in this subtopic of the review. Specifically, Nadaud [42] examined the reasons behind parents’ choices to pack lunches for their children and the role of psychological factors and perceptions related to LBFH among parents.

Both Nadaud [42] and Farris et al. [46] found that parents preferred packing food that their children liked, mainly due to concerns that school meals were perceived as not healthy or of poor quality, which their children might not eat. Also, school administrators’ interviews [42] indicated that children who brought lunches from home had more time to eat than those who ate the school lunch. Moreover, lunchtime aids and other adults, including principals and parent volunteers, play important roles in what children ate during lunch. There is a general perception that LBFH offers a wide variety of nutritional diverse foods, often influenced by the child’s cultural background. Importantly, LBFH were viewed as providing parents and children with the freedom to select and enjoy the foods they choose to bring to school.

## Discussion

Although the NSLP serves low-cost or free lunches to over 30 million children each school day [53], ∼40% of children brought lunches from home to school before the COVID-19 pandemic [15]. Despite efforts that improved the nutritional quality of school lunches and promoted NSLP participation, a substantial proportion of children still bring lunches from home. Thus, we sought to systematically review the existing research on LBFH and evaluate their food and nutritional quality.

Based on our review, 3 important findings have been identified. First, regarding food content and nutritional quality, as well as costs of LBFH, this review found that: *1*) the nutritional quality of LBFH was generally lower compared with lunches provided through NSLP. Some of the included studies specifically assessed the quality of LBFH by directly comparing it to NSLP standards, while others compared the composition of LBFH to the composition of actual lunches served at school by NSLP. LBFH were lower in essential nutrients such as calcium, dietary fiber, iron, protein, and vitamin A, while containing higher levels of carbohydrates, and saturated fat [[16], [17], [18],^34^,^40^]. Although LBFH are not required to meet the same nutrition standards as school meals, the included studies found that they often failed to meet these standards when compared. *2*) LBFH lacked several crucial food groups essential for a healthy diet when foods included in LBFH were compared with foods included in NSLP. Across all age groups, they contained fewer vegetables and less milk, but more desserts, snack foods, and SSBs compared with lunches provided through NSLP. *3*) An evaluation of cost-effectiveness revealed that nutritionally well-balanced LBFH were less cost effective after accounting for preparation time and other opportunity costs. Studies comparing the costs of LBFH to the cost of lunches served by NSLP, without accounting for meals served for free or at a reduced price to the majority of NSLP participants, found that school meals were not only nutritionally balanced but also offered at a lower price.

Second, regarding the intervention programs designed to improve the food and nutrient quality of LBFH, 2 studies were found with programs designed only for elementary school children. These interventions include programs such as *Great Taste, Less Waste, Food 2 Choose,* and *PACK-IT* employ educational approaches for children and parents to address the lack of nutrition knowledge that can prevent the selection of healthy food items for LBFH [49,52]. Neither study found significant changes in the nutritional quality of LBFH as a result of the intervention. Future research with larger sample sizes and longer durations will be necessary to explore if the strategies proposed in the study can effectively encourage parents to improve the nutritional quality of foods in LBFH. Overall, evidence remains limited, emphasizing the need for further research in this area [15,37,54]. Although not included in this review, few intervention studies conducted in preschool settings show promising results [[55], [56], [57]]. Specifically, the *Lunch is in the Bag* program, which targeted preschoolers, employed follow-up prompts using reminders as the post-intervention maintenance strategy for boosting the effectiveness of interventions to improve the nutritional quality of LBFH [58]. This approach led to a significant increase in the inclusion of whole grains and FVs in LBFH.

Third, preferences and perceptions related to nutritional quality and content of lunches vary across parents, especially those with children attending schools with lower rates of free/reduced-price lunch eligibility. There was also heterogeneity in parents’ opinions about including organic or sustainable foods in LBFH [46]. Many parents believe that school lunches are of poor quality and that their children would not eat them [42]. However, some parents may encounter various obstacles, including time constraints, limited accessibility to certain food options, and financial constraints, which can limit their ability to prepare and pack healthy lunches for their children to bring to school [59]. It is important to note that responses from parents whose children do not participate in school meal programs may be subject to bias. These parents may have limited firsthand knowledge of the actual quality and content of school meals, and their perceptions could be influenced by various factors such as their children’s subjective views, media coverage of very specific school meal-related incidences, or second-hand information from other parents and children. In addition, either a parent or child could have a specific health belief or dietary preferences that inform their decision to opt out of the school meal program, which may not be representative of the larger population of parents.

Findings from this systematic review and meta-analysis have important implications. They emphasize the need for significant nutritional improvement in LBFH, especially considering the potential adverse health outcomes and academic performance associated with the consumption of less healthy foods [60]. Given the barriers parents face in preparing lunches for their children to take to school, promoting participation in the NSLP could help ensure that more children receive nutritionally balanced and affordable lunches. Participation in NSLP not only provides children with access to meals that provide a wider range of foods and higher intakes of nutrients than LBFH [19,61] but also addresses the challenges associated with the cost and effort required for parents to pack lunches [47].

Moreover, when developing intervention programs to improve the nutritional quality of LBFH, it is crucial to consider the common barriers faced by parents. These programs should be built upon well-established behavioral theories and address barriers such as food costs, time constraints, and taste preferences [[62], [63], [64]]. In addition, barriers such as competing priorities, perishability of FVs, distance to grocery stores, and cost of specialized equipment needed to prepare fresh foods are crucial in determining the nutritional quality of LBFH of children, especially those living in low-income households [50].

Additional research is needed to explore differences in nutritional quality of LBFH and the factors driving their choice of LBFH over school lunches across gender, age, and race/ethnicity of child. This will help inform the development and implementation of intervention programs tailored to parents and children.

## Strengths and Limitations

To our knowledge, this review is the first systematic review with meta-analysis specifically focusing on LBFH to school by US school-age children, despite the existence of several review papers on school lunches [12,13]. Moreover, the comprehensive approach of this review covers a broader spectrum by addressing both quantitative and qualitative research questions, providing valuable insights into children's eating habits related to LBFH. Furthermore, the use of meta-analyses to quantitatively summarize the findings by combining data from diverse studies enhances the review’s reliability and potential for generalization, while accounting for potential biases using statistical methods.

Nevertheless, this review also has a few limitations that may impact the generalizability of the results. The major limitation is the heterogeneity found between included studies, which can be attributable to several factors.

First, the included studies employ diverse data collection methods including direct observation, 24-h recall, and digital photography. Although direct observation allows for the collection of real-time data, it might not track or capture all foods in LBFH, particularly if children consume or discard items outside of the observation period. Similarly, the 24-h recall method relies on individual memory, which can lead to inaccuracies or underreporting of certain food items. Digital photography, although useful for documenting lunch contents, may be limited by the quality and clarity of the images, potentially obscuring some food items.

In addition, the short duration of most included studies, typically less than a week, may fail to capture long-term dietary behaviors and variations in intake across different meals and days. This limitation may result in an incomplete picture of children’s overall dietary patterns and the nutritional content of their lunches over an extended period.

Another limitation is the lack of comprehensive nutrient data across all studies. Not all studies provided data on specific nutrients, such as sugar, zinc, iron, fiber, and carbohydrates, resulting in only a subset of studies being used in meta-analyses. This restricts the comprehensiveness of the findings, as they may not reflect the full nutritional profiles of LBFH. In addition, the US Department of Agriculture found that ∼20% of calories from the NSLP lunch offered is wasted (not consumed) and ∼25% of specific nutrients (vitamins A, C, and D; calcium; and potassium) are wasted [6]. Therefore, a comparison of nutrients consumed from LBFH to the nutrient content of NSLP may be biased toward finding lower nutrient intakes from LBFH if students who bring lunch from home also do not eat all of the lunch.

Furthermore, the big variation in sample sizes across the included studies makes it difficult to generalize the findings to a larger population. In addition, the geographical concentration of studies in specific locations, such as Texas and California, where only a limited number of schools or grades were involved, may not reflect the dietary habits across these states or throughout the nation. This geographical concentration also leads to a limited understanding of diverse dietary patterns influenced by the different cultural or socioeconomic backgrounds within those states.

In conclusion, this systematic review and meta-analysis provide much-needed quantitative and qualitative evidence on the quality of LBFH for school children and compare them with NSLP school lunches and nutrition standards. This review found that LBFH is less nutritionally balanced, often containing more sweets and fewer vegetables compared with NSLP lunches. With no standards in place for LBFH, the improvements to already nutritious lunches offered by NSLP will further widen this gap. In addition, findings showed the mixed effects of interventions in improving the food content and nutritional quality of LBFH for school-age children. Existing studies collected primary data from specific regions, school districts, schools, and/or age groups of children. It is important to recognize that collecting data on LBFH in a school setting remains a challenge. The findings of this review, thus, indicate the need for well-designed, nationally representative studies focused on evaluating the nutritional quality of LBFH, taking into account the sources of food, such as meals prepared and packed at home, food purchased from fast-food restaurants, leftovers from dining out, and other sources. Such research should also aim to understand the causal effect of NSLP participation to further explain the characteristics and preferences of children and their parents who prefer LBFH over NSLP meals **[**6**]**. This knowledge will be invaluable in identifying strategies to improve parent’s and children’s perceptions of NSLP and developing intervention programs tailored for both parents and children who opt for LBFH as their meal choice. Especially with many states making free meals available to all children, identifying effective ways in promoting and increasing NSLP participation can ensure more children have access to nutritionally balanced and affordable lunches.

## Author contributions

The authors’ contributions were as follows – SS, AI: conceptualized the study and designed the protocol of the systematic review; SS, ET: developed a search strategy; MC: carried out the search; SS, ET, AI: screened titles, abstracts, full-text articles and quality assessment; SS, ET: performed the data extraction and conducted meta-analysis; AI, checked the quality and correctness of extracted data; SS, wrote the first draft with critical feedback and significant revisions contributed by AI, JD; and all authors: read and approved the final manuscript.

## Conflict of interest

The authors report no conflicts of interest.

## Funding

This research was supported by funding from the Alliance for Potato Research and Education (AI). This project was also partly supported through federal funds from the USDA/Agricultural Research Service under Cooperative Agreement no. 3092-51000-058-2S (JD). The contents of this publication do not necessarily reflect the views or policies of the USDA, nor does mention of trade names, commercial products, or organizations imply endorsement by the US Government.

## References

[1] Banfield E.C., Liu Y., Davis J.S., Chang S., Frazier-Wood A.C. (2016). Poor adherence to US dietary guidelines for children and adolescents in the national health and nutrition examination survey population. J. Acad. Nutr. Diet..

[2] Ambrosini G.L. (2014). Childhood dietary patterns and later obesity: a review of the evidence. Proc. Nutr. Soc..

[3] Cullen K.W., Chen T.A. (2017). The contribution of the USDA school breakfast and lunch program meals to student daily dietary intake. Prev. Med. Rep..

[4] (2010). https://www.govinfo.gov/app/details/PLAW-111publ296.

[5] Kinderknecht K., Harris C., Jones-Smith J. (2020). Association of the healthy, hunger-free kids act with dietary quality among children in the US National School Lunch Program. JAMA.

[6] U.S. Department of Agirculture, Food and Nutrition Service, Office of Policy Support, School Nutrition and Meal Cost Study**, Final Report Volume 4: Student Participation, Satisfaction, Plate Waste, and Dietary Intakes** by Mary Kay Fox, Elizabeth Gearan, Charlotte Cabili, Dallas Dotter, Katherine Niland, Liana Washburn, Nora Paxton, Lauren Olsho, Lindsay LeClair, and Vinh Tran. Project Officer: John Endahl. Alexandria, VA: April 2019.

[7] Crepinsek M.K., Gordon A.R., McKinney P.M., Condon E.M., Wilson A. (2009). Meals offered and served in US public schools: do they meet nutrient standards?. J. Am. Diet. Assoc..

[8] Cohen J.F., Richardson S., Parker E., Catalano P.J., Rimm E.B. (2014). Impact of the new U.S. Department of Agriculture school meal standards on food selection, consumption, and waste. Am. J. Prev. Med..

[9] Gearan E.C., Monzella K., Jennings L., Fox M.K. (2020). Differences in diet quality between school lunch participants and nonparticipants in the United States by income and race. Nutrients.

[10] Gearan E.C., Fox M.K. (2020). Updated nutrition standards have significantly improved the nutritional quality of school lunches and breakfasts. J. Acad. Nutr. Diet..

[11] U.S. Department of Agriculture, Food and Nutrition Service, Office of Policy Support, School Nutrition and Meal Cost Study**, Final Report Volume 2: Nutritional Characteristics of School Meals** by Mary Kay Fox, Katherine Niland, Dallas Dotter, Liana Washburn, Patricia Connor, Laurent Olsho, and Tara Wommak. Project Officer: John Endahl. Alexandria, VA: April 2019.

[12] Cohen J.F.W., Hecht A.A., Hager E.R., Turner L., Burkholder K., Schwartz M.B. (2021). Strategies to improve school meal consumption: a systematic review. Nutrients.

[13] Cohen J.F.W., Hecht A.A., McLoughlin G.M., Turner L., Schwartz M.B. (2021). Universal school meals and associations with student participation, attendance, academic performance, diet quality, food security, and body mass index: a systematic review. Nutrients.

[14] Metcalfe J.J., Ellison B., Hamdi N., Richardson R., Prescott M.P. (2020). A systematic review of school meal nudge interventions to improve youth food behaviors. Int. J. Behav. Nutr. Phys. Act..

[15] Johnston C.A., Moreno J.P., El-Mubasher A., Woehler D. (2012). School lunches and lunches brought from home: a comparative analysis, Child. Obes.

[16] Hamilton P.C. (2001). https://researchrepository.wvu.edu/etd/1249.

[17] Doughty K.N. (2009). https://www.proquest.com/docview/305139778?pq-origsite=gscholar&amp;fromopenview=true&amp;sorucetype=Disserations%20&amp;%20Theses.

[18] Bergman E.A., Saade C., Shaw E., Englund T., Cashman L., Taylor K.W. (2014). Lunches selected and consumed from the National School Lunch Program in schools designated as HealthierUS School Challenge schools are more nutritious than lunches brought from home. J. Child Nutr. Manag..

[19] Bergman E.A., Englund T., Ogan D., Watkins T., Barbee M., Rushing K. (2016). Beverage selections and impact on Healthy Eating Index scores in elementary children’s lunches from school and from home. J. Child Nutr. Manag..

[20] Caruso M.L., Cullen KW. (2015). Quality and cost of student lunches brought from home. JAMA Pediatr.

[21] Taylor J.C., Sutter C., Ontai L.L., Nishina A., Zidenberg-Cherr S. (2019). Comparisons of school and home-packed lunches for fruit and vegetable dietary behaviours among school-aged youths. Public Health Nutr.

[22] Romo-Palafox M.J., Ranjit N., Sweitzer S.J., Roberts-Gray C., Byrd-Williams C.E., Briley M.E. (2017). Adequacy of parent-packed lunches and preschooler’s consumption compared to dietary reference intake recommendations. J. Am. Coll. Nutr..

[23] National School Lunch Program and School Breakfast Program (1995). http://www.federalregister.gov/d/95-14292.

[24] Covidence Systematic Review Software, Veritas Health Innovation, Melbourne, Australia.

[25] Basiotis P.P., Carlson A., Gerrior S.A., Juan W.Y., Lino M. (2004). The Healthy Eating Index, 1999–2000: charting dietary patterns of Americans. Fam. Econ. Nutr. Rev..

[26] US Department of Agriculture Food and Nutrition Service (2017). https://nesr.usda.gov/2015-dietary-guidelines-advisory-committee-systematic-reviews.

[27] Popay J., Roberts H., Sowden A., Petticrew M., Arai L., Rodgers M. (2006). A product from the ESRC methods programme.

[28] Lipsey M.W., Wilson D.B. (2001).

[29] Guenther P.M., Kirkpatrick S.I., Reedy J., Krebs-Smith S.M., Buckman D.W., Dodd K.W. (2014). The Healthy Eating Index-2010 is a valid and reliable measure of diet quality according to the 2010 Dietary Guidelines for Americans. J. Nutr..

[30] Cooper H., Hedges L.V., Valentine J.C. (2019). The Handbook of Research Synthesis and Meta-analysis.

[31] Neubauer B.A. (2000). https://www.proquest.com/docview/249955480?pq-origsite=gscholar&amp;fromopenview=true&amp;sourcetype=Disserations%20&amp;%20Theses.

[32] Johnson C.M., Bednar C., Kwon J., Gustof A. (2009). Comparison of nutrient content and cost of home-packed lunches to reimbursable school lunch nutrient standards and prices. J. Child Nutr. Manag..

[33] Song S., Ishdorj A., Dave J.M. (2021). Gender differences in nutritional quality and consumption of lunches brought from home to school. Int. J. Environ. Res. Public Health..

[34] Rainville A.J. (2001). Nutritional quality of reimbursable school lunches compared to lunches brought from home in elementary schools in two southeastern Michigan districts. J. Child Nutr. Manag..

[35] Conway T.L., Sallis J.F., Pelletier R.L., Powers H.S., Marshall S.J., Zive M.M. (2002). What do middle school children bring in their bag lunches?. Prev. Med..

[36] Zive M.M., Elder J.P., Prochaska J.J., Conway T.L., Pelletier R.L., Marshall S. (2002). Sources of dietary fat in middle schools. Prev. Med..

[37] Evans R.A. (2007).

[38] Lund S.M. (2008).

[39] Warren S.S. (2010). https://www.proquest.com/docview/305242033?pq-origsite=gscholar&amp;fromopenview=true&amp;sourcetype=Disserations%20&amp;%20Theses.

[40] Hur I., Burgess-Champoux T., Reicks M. (2011). Higher quality intake from school lunch meals compared with bagged lunches, Infant Child Adolesc. Nutr.

[41] Au L.E., Rosen N.J., Fenton K., Hecht K., Ritchie L.D. (2016). Eating school lunch is associated with higher diet quality among elementary school students. J. Acad. Nutr. Diet..

[42] Nadaud P. (2018).

[43] Sutter C., Taylor J.C., Nishina A., Ontai L.L. (2019). Parental and family predictors of fruits and vegetables in elementary school children’s home-packed lunches across a school week. Appetite.

[44] Farris A.R., Misyak S., Duffey K.J., Davis G.C., Hosig K., Atzaba-Poria N. (2014). Nutritional comparison of packed and school lunches in pre-kindergarten and kindergarten children following the implementation of the 2012–2013 National School Lunch Program standards. J. Nutr. Educ. Behav..

[45] Farris A.R. (2015).

[46] Farris A.R., Misyak S., Duffey K.J., Atzaba-Poria N., Hosig K., Davis G.C. (2016). Elementary parent perceptions of packing lunches and the National School Lunch Program. J. Child Nutr. Manag..

[47] O’Keefe K., Serrano E., Davis G., Cole D.A., Frisard M.I., Farris A.R. (2020). Comparison of costs between school and packed lunches. J. Child Nutr. Manag..

[48] Hubbard K.L., Must A., Eliasziw M., Folta S.C., Goldberg J. (2014). What's in children’s backpacks: Foods brought from home. J. Acad. Nutr. Diet..

[49] Goldberg J.P., Folta S.C., Eliasziw M., Koch-Weser S., Economos C.D., Hubbard K.L. (2015). Great taste, less waste: a cluster-randomized trial using a communications campaign to improve the quality of foods brought from home to school by elementary school children. Prev. Med..

[50] Blondin S.A., AlSukait R., Bleiweiss-Sande R., Economos C.D., Tanskey L.A., Goldberg J.P. (2021). Processed and packed: how refined are the foods that children bring to school for snack and lunch?. J. Acad. Nutr. Diet..

[51] Higgins J.P., Thompson S.G., Deeks J.J., Altman D.G. (2003). Measuring inconsistency in meta-analyses. BMJ.

[52] Shukaitis J., Elnakib S., Cuite C. (2021). Yumbox: a tool to improve the quality of preschoolers’ packed lunches. J. Nutr. Educ. Behav..

[53] US Department of Agriculture Food and Nutrition Service (2019). National School Lunch Program (NSLP) Fact Sheet.

[54] Ohri-Vachaspati P. (2014). Parental perception of the nutritional quality of school meals and its association with students’ school lunch participation. Appetite.

[55] Roberts-Gray C., Ranjit N., Sweitzer S.J., Byrd-Williams C.E., Romo-Palafox M.J., Briley M.E. (2018). Parent packs, child eats: surprising results of Lunch is in the Bag’s efficacy trial. Appetite.

[56] Sweitzer S.J., Briley M.E., Roberts-Gray C., Hoelscher D.M., Harrist R.B., Staskel D.M. (2010). Lunch is in the bag: increasing fruits, vegetables, and whole grains in sack lunches of preschool-aged children. J. Am. Diet. Assoc..

[57] Roberts-Gray C., Briley M.E., Ranjit N., Byrd-Williams C.E., Sweitzer S.J., Sharma S.V. (2016). Efficacy of the Lunch is in the Bag intervention to increase parents’ packing of healthy bag lunches for young children: a cluster-randomized trial in early care and education centers. Int. J. Behav. Nutr. Phys. Act..

[58] Sweitzer S.J., Ranjit N., Calloway E.E., Hoelscher D.M., Almansor F., Briley M.E. (2016). Examining how adding a booster to a behavioral nutrition intervention prompts parents to pack more vegetables and whole gains in their preschool children’s sack lunches. Behav. Med..

[59] Minaya S., Rainville A.J. (2016). How nutritious are children’s packed school lunches? A comparison of lunches brought from home and school lunches. J. Child Nutr. Manag..

[60] Martínez Steele E., Popkin B.M., Swinburn B., Monteiro C.A. (2017). The share of ultra-processed foods and the overall nutritional quality of diets in the US: evidence from a nationally representative cross-sectional study. Popul. Health Metr..

[61] Vernarelli J.A., O’Brien B. (2017). A vote for school lunches: school lunches provide superior nutrient quality than lunches obtained from other sources in a nationally representative sample of US children. Nutrients.

[62] Horning M.L., Fulkerson J.A., Friend S.E., Story M. (2017). Reasons parents buy prepackaged, processed meals: it is more complicated than “I don't have time”. J. Nutr. Educ. Behav..

[63] Zorbas C., Palermo C., Chung A., Iguacel I., Peeters A., Bennett R. (2018). Factors perceived to influence healthy eating: a systematic review and meta-ethnographic synthesis of the literature. Nutr. Rev..

[64] Moran A.J., Khandpur N., Polacsek M., Thorndike A.N., Franckle R.L., Boulos R. (2019). Make it fresh, for less! A supermarket meal bundling and electronic reminder intervention to promote healthy purchases among families with children. J. Nutr. Educ. Behav..

