# Supplementary material for: The Quality of Lunches Brought from Home to School: A Systematic Review and Meta-Analysis
Source: Adv Nutr. 2024 Jun 12;15(8):100255. doi: 10.1016/j.advnut.2024.100255 (PMC11324822; doi:10.1016/j.advnut.2024.100255)
Supplement: Multimedia component 1 [file mmc1.docx]

**Supplementary material**

1. Full database search details

**EBSCO Medline Complete (1857-present)**

1995-current

Searched on 12/6/2021 (206 results)

(MH ( Students OR Schools OR Child, Preschool OR Child OR Adolescent ) OR TI ( student* OR school children OR schoolchildren OR child OR preschool child* OR adolescent OR teen* OR youth* OR pre K* OR pre-K* OR school age OR school-age OR elementary school OR middle school OR intermediate school OR kindergarten OR high school ) OR AB ( student* OR school children OR schoolchildren OR child OR preschool child* OR adolescent OR teen* OR youth* OR pre K* OR pre-K* OR school age OR school-age OR elementary school OR middle school OR intermediate school OR kindergarten OR high school )) AND (TI ( brown bag OR brownbag OR paper bag OR Home-pack* OR lunch* brought from home OR lunch is in the bag OR NSLP OR lunch* N3 (sack OR brown bag OR pack* OR bag) ) OR AB ( brown bag OR brownbag OR paper bag OR Home-pack* OR lunch* brought from home OR lunch is in the bag OR NSLP OR lunch* N3 (sack OR brown bag OR pack* OR bag))) AND (MH ( Nutrition Assessment OR Nutritional Requirements OR Nutritive Value OR Nutrition Policy OR Food Analysis OR Feeding Behavior OR Food Quality OR Food Services OR Food Preferences OR Parents OR Program Evaluation OR Parenting OR Diet, Food, and Nutrition OR Nutritional Physiological Phenomena OR Adolescent Nutritional Physiological Phenomena OR Child Nutritional Physiological Phenomena OR Waste Products OR Diet, Healthy ) OR AB ( parent perception OR parent perspective OR perception* OR perspective* OR Nutritional quality OR food N3 (analysis OR quality OR preference*) OR feeding behavior OR Food-based standards OR food based standards OR nutritional physiological phenomena OR food waste OR healthy eating index OR healthy diet OR Dietary Reference Intakes OR DRIs OR School Meals Initiative for Healthy Children OR SMI OR school meals initiative OR cost ) OR TI ( parent perception OR parent perspective OR perception* OR perspective* OR Nutritional quality OR food N3 (analysis OR quality OR preference*) OR feeding behavior OR Food-based standards OR food based standards OR nutritional physiological phenomena OR food waste OR healthy eating index OR healthy diet OR Dietary Reference Intakes OR DRIs OR School Meals Initiative for Healthy Children OR SMI OR school meals initiative OR cost ))

**EBSCO Agricola (1970-present)**

1995-current

Searched on 12/6/2021 (159 results)

( MH preschool or preschool child or preschool children or preschool children (2-5 years) or adolescent or students or schools or child ) OR TI ( student* OR school children OR schoolchildren OR child OR preschool child* OR adolescent OR teen* OR youth* OR pre K* OR pre-K* OR school age OR school-age OR elementary school OR middle school OR intermediate school OR kindergarten OR high school ) OR AB ( student* OR school children OR schoolchildren OR child OR preschool child* OR adolescent OR teen* OR youth* OR pre K* OR pre-K* OR school age OR school-age OR elementary school OR middle school OR intermediate school OR kindergarten OR high school ) AND TI ( brown bag OR brownbag OR paper bag OR Home-pack* OR lunch* brought from home OR lunch is in the bag OR NSLP OR lunch* N3 (sack OR brown bag OR pack* OR bag) ) OR AB ( brown bag OR brownbag OR paper bag OR Home-pack* OR lunch* brought from home OR lunch is in the bag OR NSLP OR lunch* N3 (sack OR brown bag OR pack* OR bag) ) AND ( MH nutrition assessment or nutritional requirements or nutritive value or nutriture or nutrition policy or food analysis or feeding behavior or feeding behaviors or feeding behaviour or food quality or food services or food preferences or parents or program evaluation or parenting or diet, food, and nutrition or nutritional physiological phenomena or  adolescent nutritional physiological phenomena or  child nutritional physiological phenomena or waste products or  healthy diet or healthy diets or healthy eating ) OR TI ( parent perception OR parent perspective OR perception* OR perspective* OR Nutritional quality OR food N3 (analysis OR quality OR preference*) OR feeding behavior OR Food-based standards OR food based standards OR nutritional physiological phenomena OR food waste OR healthy eating index OR healthy diet OR Dietary Reference Intakes OR DRIs OR School Meals Initiative for Healthy Children OR SMI OR school meals initiative OR cost ) OR AB ( parent perception OR parent perspective OR perception* OR perspective* OR Nutritional quality OR food N3 (analysis OR quality OR preference*) OR feeding behavior OR Food-based standards OR food based standards OR nutritional physiological phenomena OR food waste OR healthy eating index OR healthy diet OR Dietary Reference Intakes OR DRIs OR School Meals Initiative for Healthy Children OR SMI OR school meals initiative OR cost )

**EBSCO ERIC (1966-present)**

1995-current

Searched on 12/6/2021 (88 results)

DE ( preschool children or adolescents or students or schools or children ) OR TI ( student* OR school children OR schoolchildren OR child OR preschool child* OR adolescent OR teen* OR youth* OR pre K* OR pre-K* OR school age OR school-age OR elementary school OR middle school OR intermediate school OR kindergarten OR high school ) OR AB ( student* OR school children OR schoolchildren OR child OR preschool child* OR adolescent OR teen* OR youth* OR pre K* OR pre-K* OR school age OR school-age OR elementary school OR middle school OR intermediate school OR kindergarten OR high school ) AND TI ( brown bag OR brownbag OR paper bag OR Home-pack* OR lunch* brought from home OR lunch is in the bag OR NSLP OR lunch* N3 (sack OR brown bag OR pack* OR bag) ) OR AB ( brown bag OR brownbag OR paper bag OR Home-pack* OR lunch* brought from home OR lunch is in the bag OR NSLP OR lunch* N3 (sack OR brown bag OR pack* OR bag) ) AND DE ( nutrition or food standards or food service or parents or program evaluation or child rearing ) OR TI ( parent perception OR parent perspective OR perception* OR perspective* OR Nutritional quality OR food N3 (analysis OR quality OR preference*) OR feeding behavior OR Food-based standards OR food based standards OR nutritional physiological phenomena OR food waste OR healthy eating index OR healthy diet OR Dietary Reference Intakes OR DRIs OR School Meals Initiative for Healthy Children OR SMI OR school meals initiative OR cost ) OR AB ( parent perception OR parent perspective OR perception* OR perspective* OR Nutritional quality OR food N3 (analysis OR quality OR preference*) OR feeding behavior OR Food-based standards OR food based standards OR nutritional physiological phenomena OR food waste OR healthy eating index OR healthy diet OR Dietary Reference Intakes OR DRIs OR School Meals Initiative for Healthy Children OR SMI OR school meals initiative OR cost )

**EBSCO CINAHL Complete (1937-present)**

1995-current

Searched on 12/6/2021 (174 results)

MH ( Students OR Students, Middle School OR Students, Elementary OR Students, High School OR Schools OR Schools, Middle OR Schools, Elementary OR Schools, Secondary OR Child, Preschool OR Child OR Adolescence ) OR TI ( student* OR school children OR schoolchildren OR child OR preschool child* OR adolescent OR teen* OR youth* OR pre K* OR pre-K* OR school age OR school-age OR elementary school OR middle school OR intermediate school OR kindergarten OR high school ) OR AB ( student* OR school children OR schoolchildren OR child OR preschool child* OR adolescent OR teen* OR youth* OR pre K* OR pre-K* OR school age OR school-age OR elementary school OR middle school OR intermediate school OR kindergarten OR high school ) AND TI ( brown bag OR brownbag OR paper bag OR Home-pack* OR lunch* brought from home OR lunch is in the bag OR NSLP OR lunch* N3 (sack OR brown bag OR pack* OR bag) ) OR AB ( brown bag OR brownbag OR paper bag OR Home-pack* OR lunch* brought from home OR lunch is in the bag OR NSLP OR lunch* N3 (sack OR brown bag OR pack* OR bag) ) AND MH ( nutrition OR Adolescent Nutrition or Food Services or parents or program evaluation or child rearing OR Nutritional Assessment OR Nutritional Requirements OR Nutritive Value OR Nutrition Policy OR Food Analysis OR Eating Behavior OR Food Quality OR Food Preferences OR Parents OR Program Evaluation OR Parenting OR Nutritional Physiology OR Adolescent Nutritional Physiology OR Child Nutritional Physiology OR Waste Products ) OR TI ( parent perception OR parent perspective OR perception* OR perspective* OR Nutritional quality OR food N3 (analysis OR quality OR preference*) OR feeding behavior OR Food-based standards OR food based standards OR nutritional physiological phenomena OR food waste OR healthy eating index OR healthy diet OR Dietary Reference Intakes OR DRIs OR School Meals Initiative for Healthy Children OR SMI OR school meals initiative OR cost ) OR AB ( parent perception OR parent perspective OR perception* OR perspective* OR Nutritional quality OR food N3 (analysis OR quality OR preference*) OR feeding behavior OR Food-based standards OR food based standards OR nutritional physiological phenomena OR food waste OR healthy eating index OR healthy diet OR Dietary Reference Intakes OR DRIs OR School Meals Initiative for Healthy Children OR SMI OR school meals initiative OR cost )

**CAB Direct**

1995-current

Searched on 12/7/2021 (163 results)

(ti:("student" OR "students" OR "school children" OR "schoolchildren" OR "child" OR "preschool child" OR "preschool children" OR "adolescent" OR "teen" OR "teenager" OR "youth" OR "youths" OR "pre K" OR "pre-K" OR "pre-kindergarten" OR "pre kindergarten" OR pre-kindergartners" OR "kindergartners" OR "school age" OR "school-age" OR "elementary school" OR "middle school" OR "intermediate school" OR "kindergarten" OR "high school") OR ab:("student" OR "students" OR "school children" OR "schoolchildren" OR "child" OR "preschool child" OR "preschool children" OR "adolescent" OR "teen" OR "teenager" OR "youth" OR "youths" OR "pre K" OR "pre-K" OR "pre-kindergarten" OR "pre kindergarten" OR pre-kindergartners" OR "kindergartners" OR "school age" OR "school-age" OR "elementary school" OR "middle school" OR "intermediate school" OR "kindergarten" OR "high school") OR de:("adolescents" OR "junior high school students" OR "high schools" OR "children" OR "high school students" OR "school children" OR "children" OR "students" OR "preschool children" OR "schools") OR id:("teenagers" OR "schoolchildren" OR "school kids")) AND (ti:("brown bag" OR "brownbag" OR "paper bag" OR "Home-packed" OR "home packed" OR "lunch brought from home" OR "lunch is in the bag" OR "NSLP" OR "sack lunch" OR "pack lunch" OR "packed lunch" OR "bag lunch" OR "bagged lunch") OR ab:("brown bag" OR "brownbag" OR "paper bag" OR "Home-packed" OR "home packed" OR "lunch brought from home" OR "lunch is in the bag" OR "NSLP" OR "sack lunch" OR "pack lunch" OR "packed lunch" OR "bag lunch" OR "bagged lunch") OR de:(“packed lunches”) OR id:(“bag lunches”)) AND (ti:(“parent perception” OR “parent perspective” OR “perception” OR “perceptions” OR “perspective” OR “perspectives” OR “Nutritional quality” OR “food analysis” OR “food quality” OR food preference” OR “feeding behavior” OR “Food-based standards” OR “food based standards” OR “nutritional physiological phenomena” OR “food waste” OR “healthy eating index” OR “healthy diet” OR “Dietary Reference Intakes” OR “DRIs” OR “School Meals Initiative for Healthy Children” OR “SMI” OR “school meals initiative” OR “cost”) OR ab:(“parent perception” OR “parent perspective” OR “perception” OR “perceptions” OR “perspective” OR “perspectives” OR “Nutritional quality” OR “food analysis” OR “food quality” OR food preference” OR “feeding behavior” OR “Food-based standards” OR “food based standards” OR “nutritional physiological phenomena” OR “food waste” OR “healthy eating index” OR “healthy diet” OR “Dietary Reference Intakes” OR “DRIs” OR “School Meals Initiative for Healthy Children” OR “SMI” OR “school meals initiative” OR “cost”) OR de:(“cost analysis” OR “food costs” OR “child nutrition” OR “nutrient intake” OR “intake” OR “nutrition” OR “perception” OR “policy” OR “nutritive value” OR “nutritional intervention” OR “parents” OR “eating” OR “feeding behaviour” OR “food preferences”) OR id:(“costing” OR “nutritional value” OR “quality for nutrition” OR “feeding behavior” OR “diet preferences” OR “taste preferences”))

**ProQuest Dissertations and Theses Global (1743-present)**

1995-current

Searched on 12/7/2021 (9 results)

AB,TI(student* OR "school children" OR schoolchildren OR child OR ("preschool children") OR adolescent OR teen* OR youth* OR ("pre katrina" OR "pre khmer" OR "pre kinder" OR "pre kindergarten") OR pre-K* OR "school age" OR school-age OR "elementary school" OR "middle school" OR "intermediate school" OR kindergarten OR "high school") AND AB,TI("brown bag" OR brownbag OR "paper bag" OR Home-pack* OR "lunch* brought from home" OR "lunch is in the bag" OR lunch* N3 (sack OR "brown bag" OR pack* OR bag)) AND AB,TI("parent perception" OR "parent perspective" OR perception* OR perspective* OR "Nutritional quality" OR food N3 (analysis OR quality OR preference*) OR "feeding behavior" OR "Food-based standards" OR "food based standards" OR "nutritional physiological phenomena" OR "food waste" OR "healthy eating index" OR "healthy diet" OR "Dietary Reference Intakes" OR DRIs OR "School Meals Initiative for Healthy Children" OR SMI OR "school meals initiative" OR cost)

1. Included studies on Healthy Eating Index Score

**Supplementary Table 1. Healthy Eating Index Score of LBFH**

| Component (Maximum Score) | Bergman et al. (2016) | Au et al. (2016) | Nadaud (2018) |
| --- | --- | --- | --- |
|  | Mean (SD) | Mean (SD) | Mean (SD) |
| **Adequacy** |  |  |  |
| Total Vegetables (5) | 1.3 (2.0) | 2 (1.6) | 2.8 (1.6) |
| Greens and Beans (5) | 0.2 (0.9) | 0.4 (1.1) | 1.9 (2.1) |
| Total Fruits (5) | 2.7 (2.3) | 2.9 (2.0) | 4.3 (1.3) |
| Whole fruits (5) | 2.7 (2.5) | 2.6 (2.3) | 4.5 (1.3) |
| Whole grains (10) | 4.7 (4.7) | 2.5 (2.7) | 5.2 (3.6) |
| Dairy (10) | 4.7 (4.4) | 5.9 (3.4) | 7.8 (2.8) |
| Total Protein foods (5) | 3.6 (1.09) | 3.7 (1.5) | 3.7 (1.4) |
| Seafood and plant proteins (5) | 1.7 (2.4) | 2.1 (2.3) | 2.9 (2.1) |
| Fatty acids (10) | 5.7 (4.3) | 4.5 (3.5) | 4.1 (3.4) |
| **Moderation** |  |  |  |
| Sodium (10) | 5.5 (4.4) | 4.3 (3.4) | 5.2 (3.2) |
| Refined grains (10) | 5.2 (4.3) | 3.7 (3.5) | 6.0 (3.3) |
| Empty calories (20) | 13.1 (6.3) | 11.4 (5.2) | 15.5 (4.4) |
| HEI Score (100) | 51.1 (15.6) | 46.1 (12.2) | 63.9 (12.2) |
